# The effect of hyperlipidemia on bone graft regeneration of peri-implantal created defects in rabbits

**DOI:** 10.1186/s40729-019-0170-x

**Published:** 2019-05-15

**Authors:** Mehmet Bugrul Tekin, Hulya Toker

**Affiliations:** 1 Oral Health Center, Kayseri, Turkey; 2 Periodontology Department, Gulhane Faculty of Dentistry, University of Health Sciences, Ankara, Turkey

**Keywords:** Peri-implant, Hyperlipidemia, Bone regeneration, Implant stability

## Abstract

**Aim:**

It is reported that hyperlipidemia affects quality and density of bone and adversely affects wound healing. The effect of hyperlipidemia on implant osseointegration and peri-implant defect regeneration has not been fully explained. The purpose of this study was to examine the effects of hyperlipidemia on the healing potential of the materials used for peri-implant bone regeneration and implant stability.

**Materials and methods:**

Twelve male, New Zealand rabbits were used in this study. Half of the rabbits were fed a 2% cholesterol diet for 8 weeks to induce hypercholesterolemia. Peri-implant defects (7 mm diameter) were created in the tibias of rabbits and placed implants (3.3 mm in diameter). This study was conducted as a split-mouth design. Animals were randomly divided into two groups: (1) hypercholesterol+autogenous graft group and hypercholesterol+xenograft group (*n* = 6), and (2) autogenous graft and xenograft groups as controls (*n* = 6). At 8 weeks after surgery, the rabbits were euthanized. During implant surgery and at 8 weeks, implant stability was measured with resonance frequency analysis (RFA values). Bone-to-implant contact (BIC) was analyzed via histomorphometric analysis.

**Results:**

Hyperlipidemic groups showed significantly lower BIC values than those of the control groups at 8 weeks (*p* < 0.05). According to baseline RFA readings, there was no significant difference between control and hyperlipidemic groups (*p* ˃ 0.05). The hypercholesterol+autogenous graft group had significantly lower RFA readings and BIC values than the hypercholesterol+xenograft group at 8 weeks (*p* < 0.05).

**Conclusion:**

Within the limitations of this study, it was found that hyperlipidemia may negatively affect the implant stability especially in the autogenous group and also, may decrease peri-implant bone regeneration. However, further studies are necessary to confirm these results more.

## Summary

These findings suggest that hyperlipidemia reduced bone graft regeneration and decreased implant stability in experimental peri-implant defects in rabbits.

Dental implant survival is mainly dependent on successful osseointegration following placement. Any alteration of this biological process may adversely affect the success rate. Also, the long-term prognosis is adversely affected by inadequate bone volume at implant sites. There are several risk factors that were defined as implant failure. One of the risks of implant’s failure depends on the systemic health of the subject (such as diabetes mellitus, osteoporosis, smoking) Recently, some authors suggested that there is a relationship between hypercholesterolemia and dental implant osseointegration [1, 2].

Hyperlipidemia is a state with an abnormal lipid profile, which is characterized by elevated blood concentrations of triglycerides, elevated levels of total cholesterol and LDL, and decreased levels of HDL cholesterol [3]. Hyperlipidemia is associated with several diseases such as atherosclerosis and osteoporosis. The National Health and Nutrition Examination Survey (NHANES III) reported that 63% of osteoporotic patients have hyperlipidemia [4]. Furthermore, in the USA, among the approximately 300,000 implants placed due to fractures every year, 5–10% exhibit impaired healing or osseointegration [5, 6]. The main mechanisms of the relationship between hyperlipidemia and bone tissue metabolism are the involved aspects of some metabolic changes, including lower bone mineral density, increase in the number of osteoclasts, and the inhibition of osteoblastic activity. However, several investigators suggested that lipid-lowering drugs, such as statins, had beneficial effects on bone metabolism and also favorable effects on statins observed on osteogenesis around implants [7].

Bone augmentation procedures stimulate or facilitate the growth of new bone into the defected site. Although autologous or autogenous bone grafts are the ideal graft material for use in periodontal and implant surgeries, its use is restricted by the limitation of graft volume available, donor site morbidity, and prolongation of the operation. Thus, various alternative materials have been used including allografts and xenografts [8–10]. Xenografts are bone grafts that are derived from a donor of different species and are accepted as an osteoconductive material [11]. It was reported that xenograft subsequently replaced newly formed bone and bone apposition around titanium implants [12]. Furthermore, Hockers et al. found that deproteinized bone mineral and autogenic bone grafts appeared to be equally well integrated into the regenerating bone around implants [13].

To the best of our knowledge, peri-implant bone graft healing in the presence of hyperlipidemia has not been studied extensively yet. However, there is only one study that has investigated the effects of hyperlipidemia on implant osseointegration in mice via implant push-in test and micro-CT analysis. In that study, Keuroghlian [1] found that high-fat diet-fed mice had significantly increased implant loss as well as decreased formation and strength of the bone-to-implant interface. Therefore, based on these unfavorable aspects of hyperlipidemia, the two hypotheses that will be tested is that high levels of cholesterol can impair bone graft regeneration around a peri-implantal defect and can decrease implant stability. Therefore, the goal of this study was to investigate the histomorphometric evaluation of xenograft and autogenous bone graft healing in high-fat diet-induced animals with peri-implant bone defects.

## Materials and methods

### Animals

Prior to the study, the protocol was approved by the Animal Ethics Committee of Cumhuriyet University Faculty of Medicine under protocol number 65202830/130. Twelve New Zealand male rabbits (6-months old), with a mean weight of 3–3.2 kg, were included in this study. The animals were maintained in individual cages at 21 °C with 12-h day/night cycles and free to access to food and fresh water.

### Induction of hyperlipidemia

Hyperlipidemia was induced on half of the animals by a 2% high-lipid diet including 95% pure cholesterol extract (Acros Organics, Geel, Belgium). Animals started on the diet 8 weeks prior to implant placement. Hyperlipidemic diet increased the total cholesterol levels fivefold at 8 weeks (Table 1), then experimental protocols were conducted. Also, blood samples were collected for the analyses of triglyceride, total cholesterol [14], and fraction (HDL and LDL) at the beginning, 4 weeks, and 8 weeks during the experiment.

**Table 1:** Biochemical parameters of study groups during hyperlipidemia induction prior to implant placement protocol (mean ± SD)

		Total cholesterol	Triglyceride	HDL	LDL
AG	Baseline	28 ± 1.1	22 ± 1.5	20 ± 2	14 ± 1.5
	4 weeks	27 ± 1.6	21 ± 1.6	19 ± 1.4	15 ± 2
	8 weeks	31 ± 1.9	25 ± 2	20 ± 1.4	18 ± 1.9
XG	Baseline	25 ± 1.4	20 ± 1.6	18 ± 1.9	17 ± 1.5
	4 weeks	30 ± 1.5	24 ± 1.5	18 ± 1.5	20 ± 1.6
	8 weeks	29 ± 1.8	19 ± 1.1	20 ± 1.4	21 ± 1.4
HPL+AG	Baseline	25 ± 2.5	23 ± 2.2	18 ± 1.2	15 ± 1.3
	4 weeks	61 ± 2.1	55 ± 1.5	38 ± 1.7	34 ± 2.6
	8 weeks	101 ± 2	90 ± 1.5	39 ± 1.5	38 ± 2.3
HPL+XG	Baseline	22 ± 1.8	18 ± 2	17 ± 2	16 ± 1.9
	4 weeks	70 ± 2.2	53 ± 1.3	36 ± 2.1	37 ± 2
	8 weeks	110 ± 1.6	94 ± 1.4	38 ± 1.6	41 ± 1.5

This study was conducted as a split-mouth design. After the hyperlipidemia induction, two implants were placed in every animal and the peri-implant defect that was restored had different treatment protocols, and the study groups were the following:

- Hypercholesterol+autogenous graft (HPL+AG) group and hypercholesterol+xenograft (HPL+XG) group (*n* = 6),
- Autogenous graft (AG) and xenograft (XG) groups as controls (*n* = 6).

### Surgical procedures

All animals were anesthetized with an intramuscular injection of ketamine (40 mg/kg, Eczacibasi Ilac Sanayi, Istanbul, Turkey) and xylazine (10 mg/kg, Bioveta a.s., Komenskeho, Chezh Republic). The surgical site was disinfected with iodine solution and shaved. A 2-cm incision was made for rising full-thickness flap in the tibia. Circumferential defect (7-mm wide and 4-mm depth) was created using trephine bur (MIS Implant Technologies, Sholomi, Israel) with a low-speed handpiece under continuous irrigation with sterile saline at both sides of the tibia. İmplant osteotomy was performed at 800 rpm and the submerged type implants (Ø3.3 mm × 8 mm, RBM surface, İmplance, Trabzon, Turkey) were placed. Circumferential defects were grafted with either AG or XG. Bovine bone (Bio-Oss, Geistlich Pharma North America, NJ, USA) with a form of granules measuring 0.25–1 mm in size was used in this study. Furthermore, the bone obtained during the defect formation was crushed in the bone crusher (Schwert, Seitingen/Oberflacht, Germany) and then implanted into defects as an autogenous graft.

The wound was sutured and postoperative antibiotic (ceftriaxone, intramuscularly (i.m.) 30 mg/kg, 3 days 1 × 1) and analgesic (carprofen, i.m. 4 mg/kg, 3 days, 1 × 1) were administered to animals to prevent postoperative infection and pain.

The animals were euthanized (by an overdose of 3% pentobarbital sodium) at 8 weeks after implant placement.

### Resonance frequency analysis measurement

The RFA was measured soon after insertion of the implant (baseline) and after 8 weeks before removal of bone blocks from tibiae, using a resonance frequency analysis device (Osstell Mentor; Integration Diagnostics AB, Göteborg, Sweden). A Smartpeg (Integration Diagnostics AB) was screwed into each implant and tightened to approximately 5 N-cm. The transducer probe was aimed at the small magnet at the top of the Smartpeg at a distance of 2 or 3 mm and held stable during the pulsing until the instrument beeped and displayed the RFA value. The measurements were taken twice in the buccolingual direction and twice in the mesiodistal direction. The mean of the four measurements was recorded as the RFA values.

### Histomorphometric analysis

Bone block sections including implants were removed and the samples were fixed in 4% neutral buffered formalin for 24 h then consecutively dehydrated using alcohol and embedded with resin (Tecnovit 7200 VLC, Heraus Kulzer GmbH, Wehrheim, Germany). The resin blocks were polymerized and sectioned in a mesiodistal plane using a cutting–grinding unit (Exakt 300 CL, Apparatbau, Norderstedt, Hamburg, Germany) in 300-μm thickness. After, the thickness of sections was carefully reduced to 40 μm using a micro-grinding system (Exakt 400 CS, Apparatbau, Norderstedt, Hamburg, Germany). Following this, the samples were stained with toluidine blue.

The histomorphometric analysis was performed by a single examiner (BT) who was masked from the samples’ identities. The images of the sections in all groups were captured by a digital camera connected to a light microscope (Olympus® CX41, Tokyo, Japan) and loaded to a computer. The image analysis software (Analysıs LS Research, Version 5.0, Olympus Soft Imaging Solutions) was used for the measurements. Bone-to-implant contact (BIC) was measured as the amount of direct contact between the bone and the implant surface. Also, the amount of BIC was recorded as micrometers.

### Statistical analysis

Statistical analyses were performed with SPSS, version 22 (IBM Corporation, New York, USA). Kolmogorov–Smirnov test was performed to test the normality of the data distribution. Mann–Whitney *U* test was used to compare groups. Wilcoxon test was performed in the intragroup analysis. The data were presented as a mean ± standard deviation and *p* < 0.05 was considered statistically significant.

## Results

The animals tolerated the surgical treatment during the experiment well. However, one animal from the hyperlipidemic group and one animal from non-hyperlipidemic group died from an infection. Also, at the end of the study, the clinical examination performed prior to sacrifice disclosed that all implants were stable and that the surrounding mucosae were clinically noninflamed.

The means of the biochemical analyses were presented in Table 2. During the study period, no significant differences were found in lipid biomarkers (TG, LDL, HDL, TC) and these levels were also higher in hyperlipidemic groups than those of the non-hyperlipidemic group (*p* < 0.05).

**Table 2:** Biochemical parameters of study groups during experiment (mean ± SD)

		Total cholesterol	Triglyceride	HDL	LDL
AG	Baseline	28 ± 2.4	22 ± 1.5	20 ± 2	14 ± 1.4
	4 weeks	27 ± 1.1	21 ± 2	19 ± 1.2	15 ± 2
	8 weeks	31 ± 1.9	25 ± 1.6	20 ± 1.5	18 ± 1.8
XG	Baseline	25 ± 1.5	20 ± 1.5	18 ± 1.9	17 ± 1.3
	4 weeks	30 ± 1.6	24 ± 1.7	18 ± 1.4	20 ± 1.4
	8 weeks	29 ± 1.5	19 ± 2.1	20 ± 1.5	21 ± 1.7
HPL+AG	Baseline	101 ± 2.5	90 ± 2.2	39 ± 1.5	38 ± 1.3
	4 weeks	105 ± 1.5	98 ± 1.2	40 ± 1.4	41 ± 2.7
	8 weeks	110 ± 1.4	100 ± 1.8	41 ± 1.7	54 ± 1.7
HPL+XG	Baseline	110 ± 2	94 ± 3.1	38 ± 2.1	41 ± 1.9
	4 weeks	113 ± 2.2	96 ± 2.2	40 ± 2	43 ± 2
	8 weeks	114 ± 1.5	101 ± 2.1	39 ± 1.4	47 ± 1.6

**Fig. 1:**
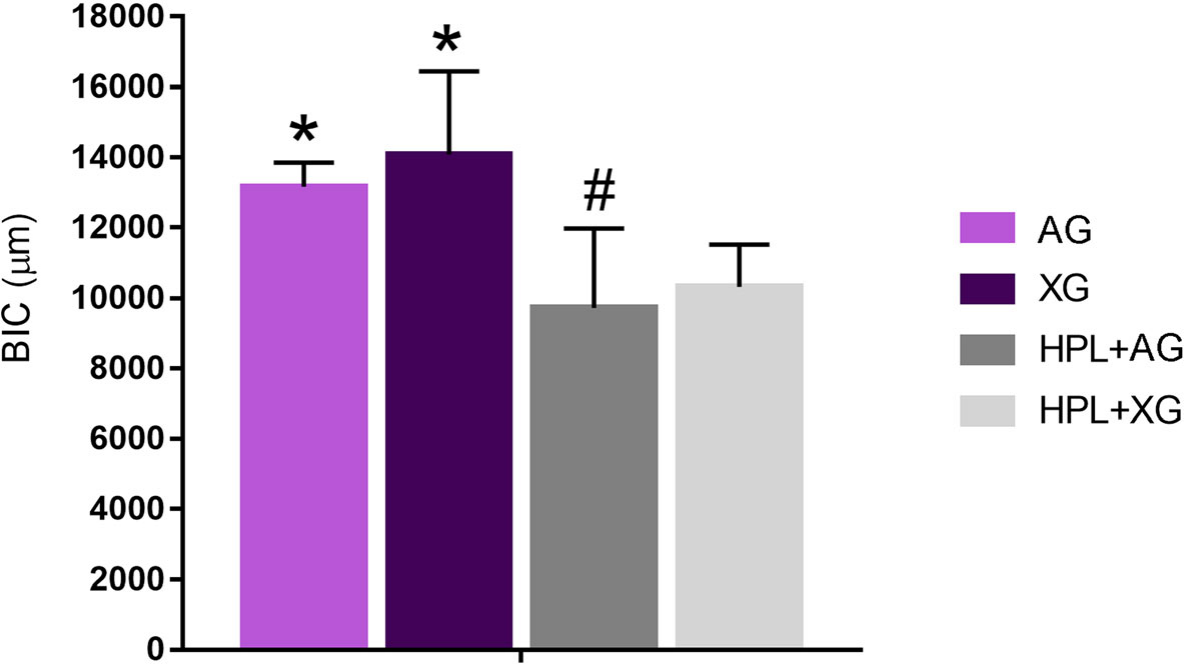
Mean BIC value of study groups at 8 weeks. *p < 0.05 versus HPL+AG and HPL+XG group, ^#^*p* < 0.05 versus HPL+XG group (*n* of each group is 5)

**Fig. 2:**
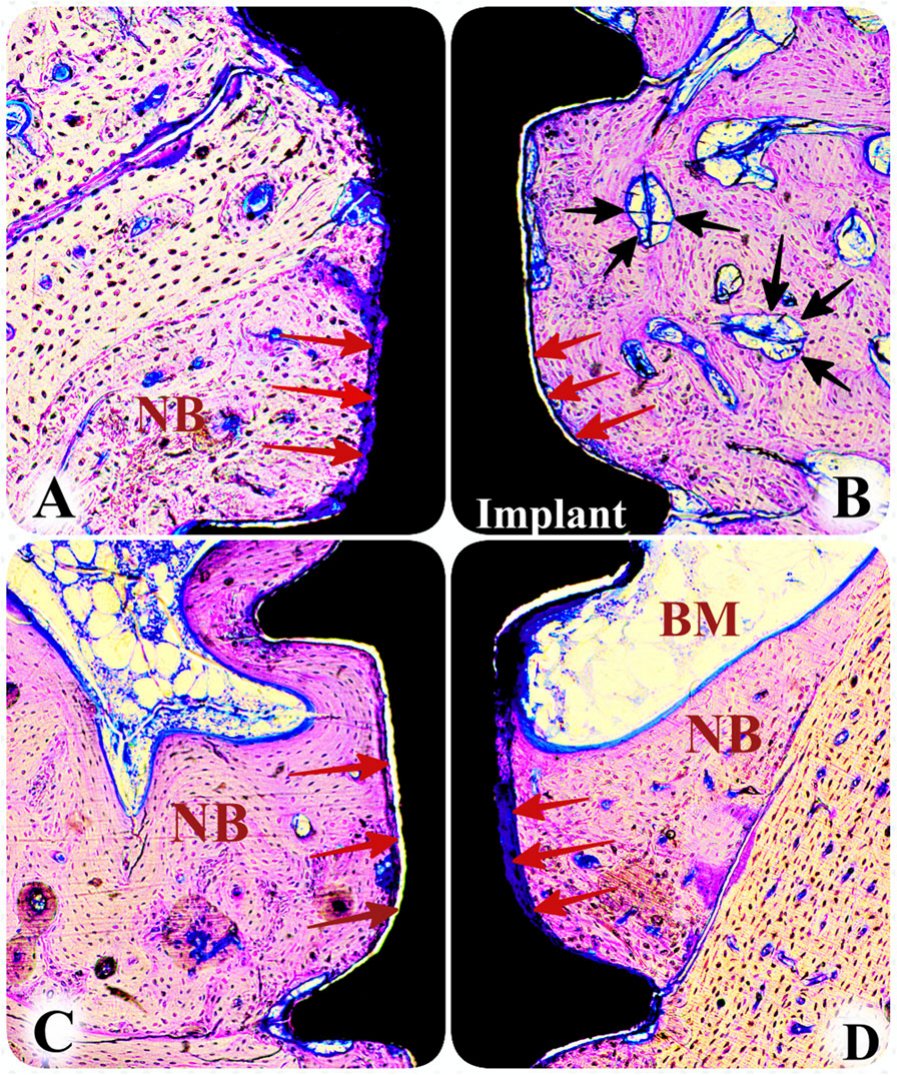
Histologic views of the study groups at 8 weeks (toluidine blue, × 10). **a** AG group, **b** XG group, **c** HPL+AG group, **d** HPL+XG group (NB new bone, BM bone marrow, red arrow indicates bone-to-implant contact, black arrow indicates residual xenograft particles)

In Fig. 1 were shown the mean value of BIC of all groups. After 8 weeks, BIC value was greater in the control groups compared to their respective hyperlipidemic groups as shown in Fig. 2 (*p* < 0.05). Also, there was a significantly higher BIC value in the HPL+XG group than those in the HPL+AG group (*p* < 0.05). However, there was no significant difference in BIC value between the AG and XG groups (*p* > 0.05).

**Fig. 3:**
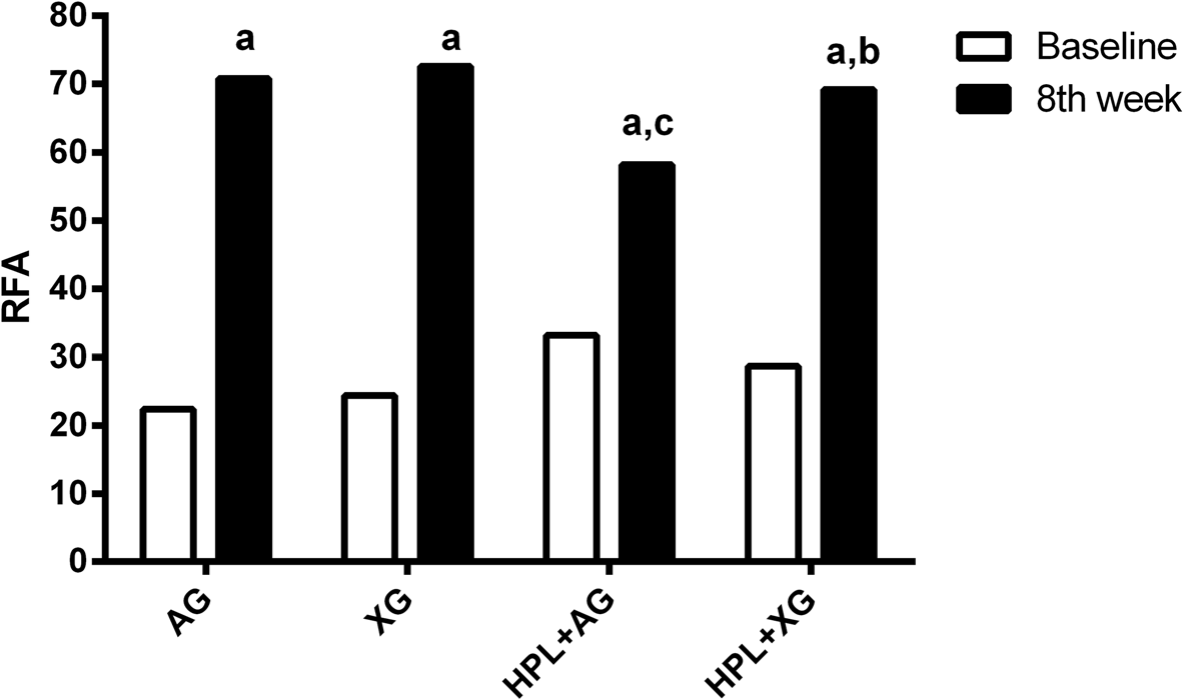
Mean and standard deviation of RFA readings in study groups at baseline and 8 weeks. ^a^*p* < 0.05 versus baseline, ^b^*p* < 0.05 versus HPL+AG group, ^c^*p* < 0.05 versus AG group (*n* of each group is 5)

RFA was measured at the time of implant placement and after 8 weeks (Fig. 3). According to intragroup comparisons, there was a significant difference in RFA readings between baseline and 8 weeks (*p* < 0.05). In all groups, RFA readings of implants were lower at baseline and were higher at 8 weeks. Also, no significant differences were found in RFA readings between AG and XG groups at 8 weeks (70.8 and 72.6, respectively) (*p* ˃ 0.05). However, in HPL+AG group, RFA readings were lower than those of the HPL+XG group at 8 weeks (*p* < 0.05). RFA readings of HPL+XG group were not different compared to XG group (*p* ˃ 0.05).

## Discussion

Although bone regeneration is an efficient process in physiological conditions, many factors such as inflammation, hormonal changes, and also, elevated serum lipid levels associated with the impaired or delayed healing process. In the present study, an initial objective of the research was to identify the effects of hyperlipidemia on bone graft regeneration around peri-implant defects in rabbits. To the best of our knowledge, this is the first study to verify the inhibition of bone graft regeneration and implant stability in peri-implantal defect under hyperlipidemic conditions. And also, we demonstrated that the amount of bone-to-implant contact and implant stability improved in HPL+XG group compared to HPL+AG group.

A wound-healing situation that is unaffected by external factors such as microbial colonization, plaque accumulation is a prerequisite for evaluating potential regenerative or augmentation treatment modalities. Therefore, in our study, the tibia of a rabbit was selected for extraoral defect model. Rabbits are commonly used for screening implant materials prior to testing in a larger animal model because they are easy to handle, and due to the size of their tibia, allowing the creation of a large peri-implant defect and the installation of conventional implants in the proximal region of the tibia [15].

In literature, different experimental protocols have been used to induce hyperlipidemia and no available threshold to confirm hyperlipidemia such as diabetic models that blood glucose levels were greater than 300 mg/dl. Earlier studies were used to induce hyperlipidemia by a different concentration or times such as a 1% cholesterol diet for 4 weeks [16], 1.5% cholesterol diet for 12 weeks [17], and 0.5% cholesterol diet for 12 weeks [18]. Also, in a recent study [19], rabbits were fed a 2% cholesterol diet for 8 weeks, as we used in our study. After 8-week feeding, we observed a fivefold increase in total cholesterol and triglyceride levels in rabbits. Furthermore, these levels lasted during the study period as shown in Table 2.

Graft resorption called biodegradation should take place at a time appropriate to the activation of new bone formation [20]. Some of the studies showed that the xenograft was slowly resorbed or was encapsulated [20–22]. In another study that was investigated histologically the simultaneous placement of endosseous implants into the sinus cavity and the surgical elevation of the sinus floor including filling the cavity with different grafting materials, they found that hydroxyapatite or natural bovine bone mineral demonstrated newly formed bone with direct contact at the implant surface [12]. Similarly, our results showed that xenograft particles were almost replaced by new bone. But, bone-to-implant contact was decreased in the hyperlipidemic group. However, there is no clinical data available in which a clinical outcome obtained following regenerative therapy of hyperlipidemic patient appears to be more unfavorable than those of the non-hyperlipidemic patients. Only one retrospective study suggested that high total serum cholesterol levels tend to increase graft failure rates whilst it did not influence implant failures. In that study, only autologous and/ or deproteinized bovine bone was used for all cases, as we used in this study [2].

Hyperlipidemia increases the risk for the generation of lipid oxidation products. Pirih’s animal study found that hyperlipidemia induces secondary hyperparathyroidism and impaired bone regeneration and mechanical strength through actions of oxidized lipids which accumulate in the subendothelial spaces of vasculature and bone [23]. Also, in a recent study, the authors evaluated the effects of hyperlipidemia on implant osseointegration in mice via micro-CT analysis [1]. They reported that HF diet-fed mice had significantly decreased bone formation and bone-to-implant contact. Interestingly, in our study, the HPL+AG group showed less BIC compared to the HPL+XG group, probably by upregulating osteoclastogenesis and suppressing mineralization. But keeping in mind that small sample size may affect these results and therefore it must be confirmed in future studies using a larger sample size.

Osseointegration and bone healing are similar processes as they both involve similar cells, hormones, and systems. Therefore, drugs or systemic diseases that affect bone healing can also decrease osseointegration [24]. In addition, the quality and quantity of the bone surrounding the implants are a critical factor to the long-term prognosis. There are several methods that have been purposed to evaluate implant osseointegration, such as the cutting torque resistance analysis and the resonance frequency analysis [25]. RFA analysis aims at providing an objective and non-invasive measurement, and also the RFA has been extensively used in experimental and clinical research [26]. However, Balshi et al. reported that RFA measurements were associated with the bone density, the location of placement, and the gender [27]. Furthermore, in a study that investigated the correlations between the RFA analysis and peri-implant bone levels in surgically created dehiscence defects and circumferential defects, they found that RFA readings were correlated to circumferential and wide-dehiscence defects but not for narrow-dehiscence defects. According to the authors, the size of surrounding bone defects was highly associated with RFA readings. Huang et al. suggested that the highest resonance frequency value was found when the implant was placed into the type I surrounding bone and in contrast, the resonance frequency of the implant with type IV bone quality was found almost fourfold less than that found in the type I model [28]. In our study, baseline RFA readings were decreased in all groups due to circumferential intra-bony defects (class 1-e, according to defect classifications of Schwarz et al.) and the wide bone marrow of the tibial bone and no significant differences among the groups [29].

In reviewing the literature, only one study has explored the effect of hyperlipidemia on implant stability in mice [1]. In that study, the most important finding was that high-fat diet-fed mice had increased implant loss and the high-fat diet group required a lower load to break the bone-to-implant contact at 4 and 8 weeks according to push-in test results. Also, in that study, there was no peri-implantal defect and all implants were inserted pristine bone of mice. However, our results showed that RFA readings were decreased in HPL+AG groups compared to controls, and RFA readings in the HPL+XG group were better results than that in the HPL+AG group. These results must be confirmed in a larger sample size because of a low sample size is the main limitation of this study.

In conclusion, within the limitations of this animal study, these findings support the hypothesis that hyperlipidemia impaired bone graft regeneration especially, autogenous graft healing with peri-implant defects and also decreased implant stability. Thus, according to our results, we confirmed the adverse effects of hyperlipidemia on implant osseointegration and peri-implant defect regeneration. However, future studies needed to investigate the mechanism of action of hyperlipidemia on graft materials in selecting appropriate graft materials for hyperlipidemic patients.
